# [Retracted] *In vitro* modulation of MMP-2 and MMP-9 in adult human sarcoma cell lines by cytokines, inducers and inhibitors

**DOI:** 10.3892/ijo.2026.5942

**Published:** 2026-09-21

**Authors:** M.W. Roomi, T. Kalinovsky, J. Monterrey, M. Rath, A. Niedzwiecki

Int J Oncol 43: 1787-1798, 2013; DOI: 10.3892/ijo.2013.2113

Following the publication of the above paper, it was drawn to the Editor's attention by a concerned reader that the gelatinase zymography data shown in Figs. 6, 10 and 14 on p. 1792, 1794 and 1795 respectively had also been included in a paper written by the same research group which had already been published in the same journal (*International Journal of Oncology*). Even though these data appeared to have been included in the two papers to show the same experimental details, given the re-use of these data in the above paper, the authors were requested to submit replacement data for these figures to be included in a corrigendum. Given the 13 years that have elapsed since the publication of these papers, the only available author was unable to verify and comply with this request; therefore, the Editor has decided that this paper should be retracted from the Journal on account of the re-use of the abovementioned data. The Editor apologizes to the readership for any inconvenience caused.

